# Association of Religious Service Attendance and Neuropsychiatric Symptoms, Cognitive Function, and Sleep Disturbances in All-Cause Dementia

**DOI:** 10.3390/ijerph20054300

**Published:** 2023-02-28

**Authors:** Katherine Carroll Britt, Kathy C. Richards, Gayle Acton, Jill Hamilton, Kavita Radhakrishnan

**Affiliations:** 1 School of Nursing, University of Pennsylvania, Philadelphia, PA 19104, USA; 2 School of Nursing, The University of Texas at Austin, Austin, TX 78712, USA; 3 Nell Hodgson Woodruff School of Nursing, Emory University, Atlanta, GA 30322, USA

**Keywords:** religion, spirituality, coping, Alzheimer’s, behavioral disturbances, older adults, protective factors, HRS

## Abstract

Commonly reported in dementia, neuropsychiatric symptoms (NPS), cognitive decline, and sleep disturbances indicate dementia progression. With the growing dementia burden, identifying protective factors that may slow dementia progression is increasingly essential. Religion and spirituality are associated with better mental and physical health, yet few studies have been reported in older adults with dementia. This study examines associations between religious service attendance and symptoms of dementia progression. Using data from the Health and Retirement Study in 2000, 2006, and 2008 and the sub-study, Aging, Demographics, and Memory Study in 2001–2003, 2006–2007, and 2008–2009, we examined the association of religious attendance with neuropsychiatric symptoms, cognitive function, and sleep disturbances among U.S. older adults aged 70 years and older with all-cause dementia (*N* = 72) using Spearman’s partial Rho correlation controlling for social interaction. Significant associations were identified for religious attendance and NPS (r_s_ (97) = –0.124, 95% CI [–0.129, –0.119], *p* < 0.0005); cognitive function, r_s_ (97) = –0.018, 95% CI [–0.023, –0.013], *p* < 0.001); and sleep disturbances, r_s_ (97) = –0.275, 95% CI [–0.280, –0.271], *p* < 0.0005). Beyond adjusting for social interaction, increased religious attendance was associated with lower NPS, better cognitive function, and fewer sleep disturbances. Clinical trials and longitudinal studies with a larger sample size examining religion and spirituality factors with dementia progression are warranted.

## 1. Introduction

A progressive condition with no cure, dementia is a public health priority [1]. The estimated U.S. cost of dementia in 2015 was $818 billion, affecting over 6 million people. The numbers are rising with the growing population of older adults, who will soon outnumber children for the first time in U.S. history [2]. Dementia disproportionately affects more historically underrepresented populations such as African American and Hispanic/Latino populations [2]. With the increase in the number of older adults and spurred by scientific advancements, older adults are living longer, creating a growing population at greater risk of Mild Cognitive Impairment (MCI) and dementia. Thus, identifying protective factors to decrease disease progression and severity is warranted.

Commonly found among older adults with MCI, with a greater prevalence among older adults with dementia [3], neuropsychiatric symptoms (NPS) may predict cognitive and functional decline [4,5]. These symptoms of apathy, aggression, depression, anxiety, agitation, delusions, and hallucinations are often challenging to manage and are associated with a higher risk of institutionalization and higher mortality [6,7]. Along with NPS, older adults with dementia experience cognitive decline over time, which affects an individual’s ability to recall information, make judgments and decisions, learn new tasks, and concentrate. Due to the progressive nature of dementia and cognitive decline, older adults with dementia often rely on others for assistance and well-being, with dementia caregivers reporting twice as many emotional, physical, and financial problems compared to caregivers of older adults without dementia [8].

With the increasing prevalence of MCI and dementia in older adults, and as lifestyle factors are a vital component of successful aging, protective factors and nonpharmacologic interventions are needed to reduce this public health burden. In precision health, identifying those at high risk of poor outcomes and disease is increasingly important to provide tailored interventions [9]. Research suggests religious and spiritual practices, which older adults find essential [10], are associated with better mental and physical health, including lower mortality, fewer depressive symptoms, and lower anxiety [11,12]. However, very few studies have examined religious and spiritual practice with health associations in older adults with dementia [13]. More research is needed to identify buffering activities that could protect against the progression of dementia. This study aimed to examine associations between religious and spiritual practice and symptoms of dementia progression, including NPS, cognitive function, and sleep disturbances.

### Theoretical Framework

**Figure 1:**
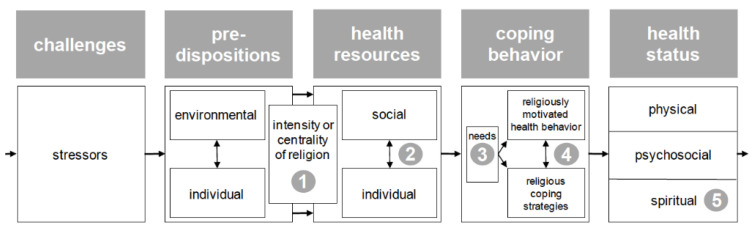
The vulnerability-stress model incorporating religiosity/spirituality (VSM-RS), which includes (1) centrality of religion, (2) R/S resources, (3) spiritual needs, (4) religious coping, and (5) spiritual well-being.

This study is strengthened by utilizing the framework of the vulnerability-stress model incorporating religiosity/spirituality (VSM-RS) [14]. This framework provides clear boundaries for religion, spirituality, and health dimensions based on religious and spiritual measures identified in health research [15] (See Figure 1). As a conceptual guide, this VSM-RS linear framework is based on established theories (i.e., transactional theory of stress and coping, diathesis-stress model). It posits that an individual’s health outcome results from an interaction between their health resources, predispositions, and coping behaviors in response to a stressor. Zwingmann and colleagues [14] suggest five religious and spiritual dimensions lie along this projected path from stressor to health outcome and include: (1) centrality of religion/spirituality, such as how vital religion/spirituality is to the individual; (2) religious/spiritual resources including individual factors and social support; (3) religious coping refers to religious and spiritual views, and understanding of stressors which may include behaviors and strategies [16]; (4) spiritual needs refer to expectations and needs that prompt an individual to find meaning, purpose, and connectedness in life [17]; and (5) quality of life/spiritual well-being refers to an individual’s sense of well-being based on life satisfaction or dissatisfaction [18]. Therefore, we use the (1) centrality of religion/spirituality dimension, which includes religious and spiritual practice and predispositions, and (5) quality of life dimension to measure health outcomes (i.e., NPS, cognition, and sleep disturbance).

## 2. Materials and Methods

### 2.1. Study Design and Participants

Secondary data analysis was conducted using established data derived from prospective cohort studies of the Health and Retirement Study (HRS) and sub-study, Aging, Demographics, and Memory Study (ADAMS). A nationally representative sample that surveyed approximately 20,000 U.S. adults aged 50 years and older, the HRS was conducted every two years beginning in 1992. The HRS study collected data on behavioral and social science variables of demographics, cognition, and health among adults living in the community and nursing homes. HRS is sponsored by the National Institute on Aging (NIA U01AG009740) and the Social Security Administration. An HRS sub-study including participants aged 70 years and older, ADAMS, was sponsored by the National Institute of Aging (grant number NIA U01AG009740) and is a high-quality sample of 1770 individuals stratified from a random subsample across five cognitive strata [19,20]. ADAMS was designed to measure the prevalence, risk factors, cost, and health outcomes of cognitive impairment and dementia among U.S. older adults. ADAMS data were collected in four collection waves over several years, beginning in wave A and ending in wave D. For the present study among persons with all-cause dementia, we examined associations of religious attendance on NPS, cognitive function, and sleep disturbances. Providing thorough evaluation and examination of a clinical diagnosis of dementia, ADAMS is the first in-home dementia assessment study of its kind across a national sample. It is especially valuable as there is no single test or measure to ascertain dementia status in large nationally represented studies. We used data from the RAND HRS Longitudinal and Fat Files in 2000, 2006, and 2008 and from ADAMS in baseline wave A in 2001–2003, wave C in 2006–2007, and wave D in 2008–2009. 

The institutional review boards approved study procedures for both data sets at The University of Michigan and Duke University Medical Center, with additional detailed descriptions and procedures from ADAMS found online [19,21,22]. Study respondents or their surrogates provided informed consent. Due to the utilization of de-identified data, the present study was granted exempt status by the institutional review board at The University of Texas at Austin. 

### 2.2. Procedures

Data were obtained from participants with dementia who completed the HRS survey in 2000, 2006, and 2008 for religious attendance and covariates used for sociodemographic, lifestyle, and behavioral factors, and the ADAMS in 2001–2003, 2006–2007, and 2008–2009 for neuropsychiatric symptoms, global cognition, and sleep disturbances. The present study analyzed the effect of religious service attendance in 2000, 2006, and 2008 on neuropsychiatric symptoms and cognition in 2001–2003, 2006–2007, and 2008–2009. 

### 2.3. Instruments 

In the ADAMS, a 3–4 h in-person structured assessment was conducted by a nurse and neuropsychology technicians trained in dementia evaluation. Evaluations were conducted in the respondent’s residential setting requiring the presence of a surrogate familiar with the medical history and everyday activities and functioning of the respondent. A neuropsychological test battery was conducted, a chronological history was taken (medical and psychiatric history, cognition and function, family history of memory impairment, behavioral symptoms), and prior neuroimaging and laboratory results were collected from each respondent’s physician [19]. A consensus panel of clinical experts evaluated and assigned a final diagnosis [23] based on published criteria: the DSM-III-R [24] and DSM-IV [25].

Following baseline data collection (wave A) in 2001–2003, approximately 18 months later, wave B was conducted, followed by wave C in 2006–2007 and wave D in 2008–2009. The mean years between data collection in waves A and C was 3.7 years. HRS provided weights for the sampling of respondents and nursing home status. 

Neuropsychiatric symptoms were measured in a structured interview with caregivers using the Neuropsychiatric Inventory (NPI). A widely accepted measure used in cognitive impairment, this scale assesses for behavioral and psychiatric behavioral disturbance presence, frequency, and severity across ten dimensions in the last month: aberrant motor behaviors, apathy, agitation/aggression, anxiety, delusions, depression, disinhibition, elation, hallucinations, and irritability [26,27]. The ADAMS study added one extra dimension that was included as a meaningful dimension for this population: sleep disturbance. The absence of a neuropsychiatric symptom was coded 0; if a symptom was present, informants reported the frequency and severity of the symptom with total scores calculated by multiplying symptom frequency and severity. The authors set a score of 4 or more as clinically and meaningful significant [28]. The validity of this scale has been reported in other studies, in addition to strong reliability [29]. Sleep disturbance was captured with three items with yes/no responses; the three-item responses were summed to create a total score (range 0–3). Sleep disturbance was operationalized as the composite number of symptoms (range 0–3) from three yes/no questions. Higher scores indicate a higher degree of disturbed sleep: (a) Do you have problems falling asleep? (b) Do you wake frequently? and (C) Do you have trouble waking too early? [30,31]. To retain partial responses for sleep disturbance, responses marked “98” (don’t know) were scored as 0 for 1–3 of the items. 

The Clinical Dementia Rating (CDR) rated participants’ cognition and functional performance from information provided by the respondent and the surrogate during evaluation [32,33]. Six domains were evaluated (memory, orientation, problem-solving, community affairs, hobbies, personal care) with a 5-point scale quantifying severity of cognitive impairment with ranges: normal (0), very mild dementia (0.5), mild dementia (1), moderate dementia (2), and severe dementia (3) [23,33]. 

Religious and spiritual activity was measured with the frequency of religious attendance. In HRS 2000, participants were asked, “Do you attend religious services?” A dichotomous response (yes/no) was recorded, and for those answering “yes”, a follow-up question was asked, “How often do you attend religious services?” Five responses range from (1) daily to (5) less than once a month. For HRS 2006 and 2008, “How often have you attended religious services during the past year?” Again, five responses range from not at all (0) to more than once per week (5). Due to the smaller sample size and response frequencies, responses were recoded into three categories: (0 = none/not at all, 1 = less than once per week, and 2 = once per week or more). The frequency of religious service attendance is a strong predictor for health outcomes, including all-cause mortality [11,12]. 

For covariates, authors adjusted for age (years), sex (0 = male, 1 = female), education in years (0–17), social contact (“frequency of getting together with people” ranging from (1) day to (6) almost never), race/ethnicity (0 = non-Hispanic White, 1 = non-Hispanic Black, 2 = non-Hispanic Other, 3 = Hispanic), and HRS wave (adjusted weights for sampling design and nursing home status). 

Demographic variables were summarized with measurements of central tendency to describe the sample: age, marital status, sex, race/ethnicity, religious preference, socioeconomic status (measured by annual household income), and education.

### 2.4. Data Management

Harmonized across cohorts, the HRS and ADAMS contain de-identified data suitable for pooled analysis. The authors used statistical analysis to test statistical assumptions evaluating the frequency, central tendency, distribution, skewness, kurtosis, outliers, missing data, and other assumption violations. For example, a respondent’s record was dropped if more than 5% of responses were missing.

### 2.5. Statistical Analyses

Baseline sociodemographic data, religious attendance, neuropsychiatric symptoms, cognition, and sleep disturbance were analyzed for all-cause dementia reporting means (SD), medians, and ranges. To examine associations between religious attendance and NPS, cognitive function, and sleep disturbance adjusted for social interaction, we calculated a bootstrapped partial Spearman’s Rho correlation using IBM SPSS Statistics (Version 25). Power was ensured with a ratio using at least 20 data points per predictor [34]. *p* values were adjusted (*p* < 0.0167) using the Bonferroni correction for accounting for multiple comparisons. All analyses were weighted and adjusted [22] to account for the complex sampling design (stratification, clustering, nonresponse) of the HRS and ADAMS studies. 

The frequency of religious attendance and social interaction were reverse-coded. Marital status was coded (0 = single, 1 = married or partnered, 2 = divorced, separated, 3 = widowed). After variables were coded correctly, data were analyzed for missing data, errors, and multicollinearity. The frequency distribution of all categorical variables and mean, standard deviation, and percentiles of all continuous variables were obtained. Cases were weighted in SPSS by HRS respondent and nursing home weight.

## 3. Results

Individuals who completed the HRS baseline data and were followed up on in ADAMS in either wave A, C, or D with a diagnosis of all-cause dementia totaled 374. After further excluding participants with missing (*N* = 296) or not assessed/ascertained data (*N* = 6) on primary study variables (i.e., frequency of religious attendance, NPS, sleep disturbance), the final sample for this study was 72 older adults.

### 3.1. Description of Sample

Participants ranged from 73–100 years with a mean age of 84.23 years, and a mean education of 10.26 years; 76.5% were non-Hispanic White, 64.5% were female, 86.6% were living in the community, 62.3% were widowed, and 64.2% were Protestant (see Table 1). The importance of religion varied across race/ethnicity: non-Hispanic White (59.4%), non-Hispanic Black (96.7%), and Hispanic (100%) participants found religion very important (see Table 2). Approximately 41.9% of non-Hispanic White participants attended religious services at least once a week compared to 36.8% of non-Hispanic Black participants and 67.3% of Hispanic participants. Examining differences across sex, males held religion as very important (75.5%) versus females (64.3%), with both holding similar percentages of attending religious services at least once a week (39.7% versus 43%) (see Table 3). 

### 3.2. Associations of Religious Attendance with Neuropsychiatric Symptoms, Cognitive Function, and Sleep Disturbances

Data did not meet regression analysis assumptions due to nonnormal distribution, nonlinearity, and heteroscedasticity. Therefore, a nonparametric correlation analysis was conducted using bootstrapped partial Spearman’s Rho for all three outcomes: NPS, cognitive function, and sleep disturbance. Controlling for social interaction, an increase in the frequency of religious attendance was significantly associated with lower NPS *r_s_* (97) = –0.124, 95% CI [–0.129, –0.119], *p* < 0.0005, better cognitive function, *r_s_* (97) = –0.018, 95% CI [–0.023, –0.013], *p* < 0.001, and lower sleep disturbance, *r_s_* (97) = –0.275, 95% CI [–0.280, –0.271], *p* < 0.0005 (see Table 4). 

## 4. Discussion

Our findings support significant associations between the frequency of religious attendance and fewer neuropsychiatric symptoms and sleep disturbances and better cognitive function in U.S. older adults living with dementia. Since this analysis was cross-sectional, it is unclear if religious attendance is protective, but associations are significant. Informed by the VSM-RS framework [14], our findings suggest that older adults facing a stressor of dementia use their predisposition for and resources of religious attendance, which support better health outcomes. Additional studies examining these associations over time will further inform this relationship. There is the potential for religious attendance to play a protective role for those facing a progressive illness such as dementia and, therefore, to possibly slow decline. With the growing dementia burden and the absence of a present cure, identifying protective factors to slow decline could lower the economic burden of dementia and decrease caregiver stress. With dementia caregivers reporting more significant problems than non-dementia caregivers [8], a non-pharmacologic solution to reduce dementia caregiver burden is greatly needed and should be examined in future studies. Even with the small effect sizes of associations identified in this study, a small improvement could be clinically meaningful, considering the increasing number of older adults being diagnosed with dementia prompted by the aging of the baby boomer generation.

Our findings indicate that religious attendance was significant beyond social interaction. There are many possible mechanisms through which religious attendance is associated with fewer NPS and sleep disturbances and better cognitive function. Religious and spiritual practices improve mental health [35,36,37], as they are associated with decreased psychological distress (i.e., anxiety, depression, anger) and increased positive psychological resources (i.e., hope, optimism, meaning) [37,38,39]. Religious and spiritual practices promote coping through stress reduction and provide a social support network with resources [11,40]. Additionally, religion and spirituality promote healthier behaviors (i.e., lower smoking and lower alcohol consumption) and enhance psychosocial well-being [41]. Religious and spiritual practices are lifestyle aspects that serve as a vital component of successful aging and may help reduce one’s risk of developing a cognitive condition [42]. Studies support the mediating effects of psychological resources on developing disease and functional decline in older adults [43,44]. Some studies indicate that leisure activities, education, and occupational exposure are associated with reduced dementia risk and slower cognitive decline rates in older adults by contributing to cognitive reserve [45,46,47]. Cognitive reserve may mediate the impact of brain pathology on the clinical manifestation of that pathology by providing more efficient brain network utilization, thus compensating for disrupted social processing networks [45]. Religious and spiritual behavior and practice may contribute to cognitive reserve through cognitively stimulating activity [48]. More studies examining the biological mechanisms by which religious attendance is associated with fewer dementia progression symptoms are needed.

Our results suggest that Hispanic and female participants report a higher frequency of religious attendance. Other studies reported these findings stating that Hispanic populations and females are more religious than non-Hispanic populations and males [49,50]. The importance of religion was highest among non-Hispanic Black, Hispanic, and male participants. This is similar to other research supporting the greater importance of religion among historically underrepresented populations compared to non-Hispanic White populations [51] yet differs from further research reporting that U.S. females are more likely to report religion as very important compared to U.S. males [52]. Historically underrepresented populations find salience in religion and spirituality for coping within their communities as they face persistent health and social inequities [53]. These populations and females have the highest prevalence of Alzheimer’s disease and related dementias, with minority (non-White) ethno-racial groups estimated to make up 45% of the U.S. population of adults aged 65 years and older in 2060 [54,55]. Because these groups find importance in religion and spirituality, exploring the potential of religious attendance to slow dementia decline in these at-risk populations could decrease disparities. Including non-Hispanic Black, Hispanic, and female participants in future studies is warranted to examine associations with the potential to inform public health interventions to support religious and spiritual practices of those at greater risk of decline.

Our study findings are consistent with previous publications [56,57]. In a longitudinal study over 12 months in Italy, Coin et al. [56] found that older adults with Alzheimer’s disease-related dementia (*N* = 64) who reported higher religiosity (i.e., frequency of prayer, religious attendance, and reading religious materials) and spirituality had slower behavioral and cognitive decline compared to those with lower religiosity and spirituality. Among 28 older adults with mild Alzheimer’s disease-related dementia, McGee et al. [57] reported that negative religious coping (i.e., religious struggles and doubts) was associated with behavioral and psychological expression frequency. To the best of our knowledge, this is the first study to show associations between religious service attendance and NPS, including sleep disturbances, in an all-cause dementia sample in the U.S. 

Other studies report conflicting findings on religious and spiritual practices with cognitive function. For example, among 70 older adults in Canada with dementia, Kaufman et al. [58] report that private religious activities predicted slower cognitive decline over 2–3 years. However, public religious activities were not associated with cognitive decline. In South Korea, Jung and colleagues examined associations in older adults with Alzheimer’s disease-related dementia (*N* = 325) and found religiosity was related to cognitive function. However, Ritchie et al. [59] conducted a longitudinal study in the U.K. among cognitively healthy older adults (*N* = 550) and found no association between religious service attendance and cognitive function.

Further, in a systematic review examining religious and spiritual factors with cognition among older adults, Hosseini et al. [60] report that 82% of the included studies (*N* = 17) reported a positive association between religious and spiritual activity and cognitive function. These findings suggest that public and private religious and spiritual practice may protect against cognitive decline. However, previous studies have not controlled for social interaction or reported loss of significance after adjusting for it with religious attendance [48]. 

Very few studies have been conducted on religious involvement and sleep outcomes [35,36,37]. Those identified reported a protective influence, suggesting that those who engage in religious practices exhibit healthier sleep patterns than those who do not [61]. In addition, Hill et al. [62] report an inverse association between religious attendance and sleep disturbance among cognitively healthy older Mexican American participants, controlling for chronic diseases including diabetes, hypertension, stroke, and heart attack. However, to our knowledge, there are no identified studies examining religious and spiritual practices on sleep among older adults with dementia. Our findings suggest that more research is needed to explore these associations over time to determine whether religious attendance may be a protective factor against sleep disturbances. 

The sample in this study is representative of the U.S. adult population; sampling weights created by the original study investigators were used, which adds to the generalizability of the findings. However, as our results are cross-sectional, additional studies are needed to examine associations over time to identify the potential effect of religious and spiritual practices on dementia progression. In addition, this study utilized a small sample, and future studies are needed using larger, religiously diverse samples to validate the present findings. Repeating the study in a larger sample size will allow additional inclusion of essential covariates in the analysis (i.e., age, income, education, sex) to determine if religious attendance remains significant with dementia symptoms of progression after adjusting for other important factors. Additionally, religious attendance was captured with one self-reported item, addressing a single dimension of religious involvement; additional items of religious involvement would increase the methodological rigor of studies focusing on religious and spiritual life. The current sample was predominantly Protestant and Catholic. Because the religious landscape in the U.S. is shifting [63], future studies should include participants from various religious backgrounds, including those who do not identify as religious. Differentiating health outcome associations with specific religious affiliations could further increase our understanding of the possible mechanisms by which religion affects health, as denominations differ in their religious teachings and practices. Additionally, historically underrepresented populations who find religion and spirituality important and are at greater risk of developing dementia should be included [8].

## 5. Conclusions

For those who find religion and spirituality important, it appears religious and spiritual practices have the potential to improve mental and physical health outcomes and reduce the risk of cognitive impairment [60,64,65,66]. However, more studies are needed to examine these associations over time in a large sample size. Most published studies have examined health associations with religious and spiritual practices in advanced illnesses such as cancer, but a large gap remains in dementia. Studying these associations over time using a diverse religious and historically underrepresented population is warranted, as these populations report finding cultural salience in religion and spirituality and are at greater risk of developing dementia. In addition, designing and testing tailored solutions supporting personal preferences of, and cultural sensitivity for, older adults with dementia is warranted to support person-centered care and well-being.

## Figures and Tables

**Table 1:** Description of Sample (*N* = 72).

Demographics	Mean (SD), Range
Age	84.23 (5.729) 73–100
Education	10.26 (4.267), 0–17
Income	25,913.67 (27,714.26), 2400–138,514.88
Cognitive Rating	1.169 (0.5316), 0.0–3.0
NPS scores	4.87 (8.747), 0–54
Sleep Disturbances	0.71 (0.982), 0–3
	Unweighted Count (N = 72) ^1^ (%)
Race/Ethnicity	
Non-Hispanic White	50 (76.5)
Non-Hispanic Black	19 (19.8)
Non-Hispanic Other	0 (0)
Hispanic	3 (3.7)
Sex	
Females	45 (64.5)
Males	27 (35.5)
Proxy	
Yes	7 (7.9)
No	65 (92.1)
Living Arrangements	
Community	62 (86.6)
Nursing Home	10(13.4)
Marital Status	
Single	0 (0)
Married, partnered	23 (33.2)
Divorced, separated	4 (4.5)
Widowed	45 (62.3)
Religious Preference	
Protestant	46 (64.2)
Catholic	19 (27.1)
Jewish	4 (5.5)
None/no preference	2 (2.6)
Other	1 (0.6)
Importance of Religion	
Not too important	6 (10.1)
Somewhat important	15 (21.6)
Very important	51 (68.3)
Religious Attendance	
Never or not at all	18 (26.5)
Less than once a week	23 (31.6)
At least once a week	31 (41.8)

Note: ^1^ Table contains raw counts and survey-weighted: means, standard deviations, median, ranges, and percentages; therefore, percentages may not sum to 100. Cognitive rating: higher number indicates greater impairment. NPS = neuropsychiatric symptoms; SD = standard deviation.

**Table 2:** Religious Frequencies Across Race/Ethnicity.

Variables	Non-Hispanic White	Non-Hispanic Black	Non-Hispanic Other	Hispanic
	*N ^* (%)	*N ^* (%)	*N ^* (%)	*N ^* (%)
Importance of Religion				
Not too important	6 (13.2)	0	0	0
Somewhat important	14 (27.4)	1 (3.3)	0	0
Very important	30 (59.4)	18 (96.7)	0	3 (100)
Frequency of Religious Attendance				
Never	14 (28.5)	3 (17.8)	0	1 (32.7)
Less than once a week	14 (29.6)	9 (45.5)	0	0
At least once a week	22 (41.9)	7 (36.8)	0	2 (67.3)

Note: ^ Table contains raw counts and survey-weighted means, standard deviations, median, ranges, and percentages; therefore, percentages may not sum to 100.

**Table 3:** Religious Frequencies of Females and Males.

Variables	Females	Males
	*N ^* (%)	*N* ^ (%)
Importance of Religion		
Not too important	3 (7.5)	3 (14.8)
Somewhat important	11 (28.2)	4 (9.6)
Very important	31 (64.3)	20 (75.5)
Frequency of Religious Attendance		
Never	13 (30.3)	5 (19.7)
Less than once a week	13 (26.7)	10 (40.6)
At least once a week	19 (43)	12 (39.7)

Note: ^ Table contains raw counts and survey-weighted means, standard deviations, median, ranges, and percentages; therefore, percentages may not sum to 100.

**Table 4:** Correlation Analysis Results.

Predictor	Controlled For	NPS	Cognitive Rating ^	Sleep Disturbances
	Variable	*r* (CI)	*r* (CI)	*r* (CI)
Religious Attendance	Social interaction	–0.124 (–0.129, –0.119) *	–0.018 (–0.023, –0.013) **	–0.275 (–0.280, –0.271) *

Note: ^ Cognitive Rating: higher number indicates more impairment. *r* = correlation; CI = Confidence Interval; NPS = neuropsychiatric symptoms. * *p* < 0.0005, ** *p* < 0.001.

## Data Availability

The data utilized in this study are publicly available at https://hrs.isr.umich.edu. (accessed on 2 November 2021).
